# Effect of malaria infection on hematological profiles of people living with human immunodeficiency virus in Gambella, southwest Ethiopia

**DOI:** 10.1186/s12878-017-0072-1

**Published:** 2017-02-02

**Authors:** Tsion Sahle, Tilahun Yemane, Lealem Gedefaw

**Affiliations:** 1 Department of Clinical Laboratory, Gambella Hospital, Gambella, Ethiopia; 2 Department of Medical Laboratory Science and Pathology, Jimma University, Jimma, Ethiopia

**Keywords:** Malaria, Anemia, HIV, Gambella

## Abstract

**Background:**

Malaria and human immunodeficiency virus are the two most devastating global health problems causing more than two million deaths each year. Hematological abnormalities such as anemia, thrombocytopenia and leucopenia are the common complications in malaria and HIV co-infected individuals. The aim of this study was to determine the effect of malaria infection on hematological profiles of people living with HIV attending Gambella Hospital ART clinic, Southwestern Ethiopia.

**Objective:**

To determine the effect of malaria infection on hematological profiles of people living with HIV attending Gambella Hospital ART clinic, Southwestern Ethiopia.

**Methods:**

A facility based comparative cross-sectional study was conducted from May 25 to November 11, 2014 in Gambella Hospital. A total of 172 adult people living with HIV (86 malaria infected and 86 malaria non-infected) participants were included in the study. Demographic, anthropometric and clinical data were collected. Venous blood samples and stool specimen were collected for laboratory analysis. Microscopic examination of peripheral blood films was done for detection of malaria parasites. Descriptive statistics, student T- test, bivariable and multivariable analyses were performed using SPSS V-20. Statistical significance was set at *p* < 0.05.

**Results:**

A total of 172 adult people living with HIV were included in the study. The prevalence of anemia, thrombocytopenia and leucopenia in malaria and HIV co-infected participants were 60.5%, 59.3%, and 43.0%, respectively. Resident (AOR: 4.67; 95% CI: 1.44, 15.14), malaria infection (AOR: 2.42; 95% CI: 1.16, 5.04) and CD_4_^+^ count were predictors for anemia. A predictor for thrombocytopenia was malaria infection (AOR: 9.79; 95% CI: 4.33, 22.17). Malaria parasitic density (AOR: 0.13; 95% CI: 0.03, 0.57) and CD_4_^+^ count (AOR: 4.77; 95% CI: 1.23, 18.45) were predictors of leucopenia.

**Conclusions:**

Findings suggest that the prevalence of anemia and thrombocytopenia were significantly higher in the malaria and HIV coinfected participants than the HIV mono-infected participants. Mean values of hematological profiles were significantly different in the two groups. Future prospective studies with larger sample size from other settings are needed to substantiate the findings.

## Background

Hematological abnormalities are the common complications in malaria infection and they play a major role in malaria pathophysiology. These changes involve the major cell lines, such as red blood cells, leucocytes and thrombocytes and the abnormalities such as anemia, thrombocytopenia and leukocytosis or leucopenia [1–5]. Hematological abnormalities such as anemia [6], neutropenia [7], and thrombocytopenia [8] are commonly reported abnormalities associated with HIV infection. HIV has effects on the systemic inflammatory response, causing activation and/or apoptosis in a variety of immune cells as well as elevated levels of proinflammatory cytokines and chemokines in plasma and lymph nodes. This immune activation is also a potential means by which HIV affects the disease course and outcome in other infections, such as malaria [9].

Malaria and HIV are the two most devastating global health problems of our time, causing more than two million deaths each year [10, 11] and greatest medical challenges facing Africa today [12]. Both malaria and HIV are diseases of poverty and can exert further poverty by affecting young adults in the work force, and this contributes to less productivity in the development of local economy [9].

Both malaria and HIV can cause hematological abnormalities independently. Those hematological abnormalities: anemia, thrombocytopenia and leucopenia have been documented as strong, independent predictors of morbidity and mortality in malaria co -infected HIV positive individuals than mono infected HIV positive individuals.

Beside this HIV infection is associated with a twofold higher risk of severe malaria in adults, and a six to eightfold increase in the risk of death [13].

Immunological complications are common in HIV infection, besides these hematological abnormalities have been documented as strong independent predictors of morbidity and mortality in HIV-infected individuals [14]. Although from hematological abnormalities cytopenia is the most frequent one but it is rare in the early stages of HIV infection [15]. Although malaria and HIV are known to be the most common public health problems in Ethiopia [16], yet limited study has been done, which evaluates the extent of hematological changes in malaria and HIV co-infected individuals particularly in this study area. Therefore, this study was intended to determine the effect of malaria infection on hematological profiles of people living with HIV attending Gambella Hospital ART clinic, Southwestern Ethiopia.

## Methods

### Study area and study period

A facility based comparative cross sectional study was conducted in the Gambella Hospital from May 25 to November 11, 2014. Gambella Hospital is located in Gambella Town which is located 777 Km Southwest of Addis Ababa, Ethiopia. Gambella Hospital is the only hospital in the region and offers services for nearly 200,000 people.

All HIV positive adults attending in the Gambella Hospital ART Clinic for routine follow up and/or treatment during the study period and willing to participate were included consecutively. HIV positive adults on any anti-malarial treatment during the study period were excluded from the study.

The sample size was calculated by using a statistical formula for comparison of two populations mean obtained from previously conducted study in Gondar University Hospital, Ethiopia [17]. Accordingly, a total of 172 (86 malaria positive and 86 malaria negative) HIV positive participants were included in the study.

### Data collection and processing

The participant socio-demographic and anthropometric data were collected using structured questionnaire and clinical data of each participant were collected from the existing ART logbook by the clinician who work in ART clinic.

Four ml of venous blood sample was collected using an EDTA containing vacutainer tube from each participant for laboratory investigation. For the detection of malaria both thick and thin blood films were prepared, stained with 10% Giemsa solution and examined with a light microscope. The malaria parasitic density was calculated by counting the number of parasites in thick blood film against 200 or 500 WBCs. The number of parasites per μl of blood was calculated [18].

The remaining blood sample was used for hematological analysis (CBC) and for the CD_4_^+^ lymphocyte count. Hematological parameters: red blood cell count (RBC), hemoglobin (Hgb), hematocrit (HCT), mean cell volume (MCV), mean cell hemoglobin (MCH), mean cell hemoglobin concentration (MCHC), total white blood cell count (WBC), differential neutrophil (NEUT), MID (Eos, Bas and Mon), differential lymphocyte count (LYM) and platelet count (PLT), were determined using the automated blood cell analyzer CELL DYNE 1800® (Abbott Laboratories Diagnostics Division, USA). CD_4_^+^ lymphocyte count was assayed using the BD FACS® COUNT (Becton Dickenson California, USA). Anemia, thrombocytopenia and leucopenia were defined as; Hgb <12 g/dl for female and Hgb <13 g/dl for male [19], platelet count < 150 × 10^9^/l and WBC count < 4.0 × 10^9^/l, respectively [20]. Stool specimen was collected and examined directly using both wet mount smear preparations and formol-ether concentration technique. To get reliable data training was given for data collectors. To assure the quality of the laboratory data standard operating procedure for each test was followed. Reagents were checked for their expiry date and prepared according to the manufacturer’s instruction. Quality control procedures were performed daily according to the laboratory’s protocol. Control samples were used for hematology analyzer and for the FACS count machine. Malaria slides were checked by two experienced laboratory technologist.

### Data analysis and interpretation

The data were checked for completeness before analysis. The descriptive statistics was used to see the distribution of the socio-demographic, anthropometric and clinical characteristics of the participants. For the continuous variables mean, standard deviation and 95% confidence interval were determined in each group. Student’s t - test was used to compare the mean value of hematological parameters between malaria infected and non-infected participants. Multivariable logistic regression was used to test the degree of association between dependent and independent variables. The variables with *p*-value ≤ 0.25 in bivariable analysis were nominated for multivariable analysis. All variables with *p*-value < 0.05 were considered as statistically significant. Data were analyzed using SPSS Version 20 (IBM Corporation, Chicago, USA) software for windows.

## Results

### General characteristics of the study participants

In a total of 172 participants with the mean age of 31.95 (±7.6) years, the majority were females (60.5%) with normal BMI (68.6%) and on HAART medication (82.6%). Among malaria and HIV infected participants 81 (94.2%), 3 (3.5%) and 2 (2.3%) of study participants were infected with *P. falciparum, P. vivax* and mixed infection, respectively. The malaria parasitic density ranges from 110 to 179,705 with a median of 4134 parasites/μl.

Of the total study participants, 16 (9.3%) had opportunistic infections. From these; four participants had tuberculosis, three participants had pneumocystis pneumonia and the other three participants had herpes zoster virus while the remaining six study participants had oesophageal candidiasis, tinea capitis, cryptococcal meningitis each account for two participants. The overall prevalence of intestinal parasites was 7.6%. *Giardia lamblia* ten (76.9%) was the most frequent parasite, followed by *Entamoeba histolytica/dispar* one (7.7%), *Hook worms* one (7.7%) and *Ascaris lumbricoides* one (7.7%).

### Hematological profiles of HIV and malaria co-infected participants

**Fig. 1:**
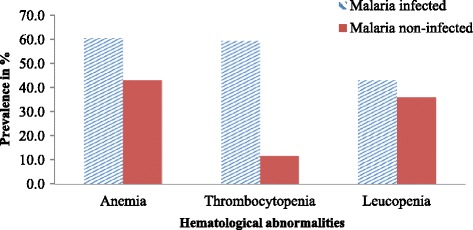
Prevalence of hematological abnormalities in PLWHA at Gambella Hospital, Southwest Ethiopia, May 25 to November 11, 2014

From a total of 172 participants, 89 (51.7%) had anemia, 61 (35.5%) had thrombocytopenia, and 68 (39.5%) had leucopenia. Among malaria and HIV co-infected study participants 52 (60.5%), 51 (59.3%), and 37 (43.0%) had anemia, thrombocytopenia and leucopenia, respectively. From malaria non-infected study participants 37 (43.0%), 10 (11.6%) and 31 (36.0%) had anemia, thrombocytopenia leucopenia, respectively. A statistically significant difference was observed in the prevalence of anemia (*P* = 0.022) and thrombocytopenia (*P* < 0.001) between the two groups (Fig. 1).

There was a significant mean difference with respect to Hgb, HCT, lymphocyte, neutrophil, and platelet values. However, no significant difference observed in values of RBC, MCV, MCH, MCHC, WBC, MID and CD_4_^+^ count (Table 1).

**Table 1:** Comparison of hematological profiles between malaria infected and malaria non- infected participants

Hematological Profiles	Malaria infected Mean ± SD	Malaria non- infected Mean ± SD	t- value	*P*-value
RBC (×10^12^/L)	3.88 ± 0.69	4.08 ± 0.66	−1.914	0.057
Hgb (g/dL)	11.77 ± 1.80	12.69 ± 2.03	−3.120	0.002
HCT (%)	34.60 ± 5.44	37.23 ± 5.59	−3.116	0.002
MCV (FL)	88.99 ± 10.86	91.10 ± 12.33	−1.190	0.236
MCH (pg)	30.53 ± 3.38	31.36 ± 4.12	−1.443	0.151
MCHC (g/dL)	34.08 ± 1.38	33.74 ± 3.08	0.921	0.359
WBC (×10^9^/L)	4.66 ± 2.19	5.09 ± 3.57	−0.944	0.346
LYM (%)	35.76 ± 14.24	48.17 ± 12.95	−5.974	<0.0001
MID (%)	14.42 ± 7.81	13.89 ± 22.80	0.204	0.838
NEUT (%)	49.11 ± 17.41	40.34 ± 12.03	3.842	<0.0001
PLT (×10^9^/L)	148.72 ± 77.91	236.88 ± 75.02	−7.559	<0.0001
CD_4_^+^ (cell/μl)	434.36 ± 292.41	460.63 ± 230.24	−0.655	0.513

*SD- Standard deviation, RBC-Red blood cell, HCT-Hematocrit, Hgb-Hemoglobin, MCV-Mean cell volume, MCH-Mean cell hemoglobin, MCHC-Mean cell hemoglobin concentration, WBC-White blood cell, LYM-Lymphocyte, MID - Average of basophils, eosinophil and monocyte, NEUT-Neutrophil, PLT- Platelets and CD_4_^+^-cluster of differentiation

### Factors associated with abnormal hematological profiles in PLWHA

Malaria infected study participants were two times more likely to be anemic than malaria non-infected study participants (AOR: 2.42; 95% CI: 1.16, 5.04) (Table 2). Study participants who were malaria infected had ten times more likely to be thrombocytopenic than malaria none infected study participants (AOR: 9.79, 95% CI: 4.33, 22.17) (Table 3). Malaria parasitic density ≥10,000 parasites/μl (AOR: 0.13; (95% CI: 0.03, 0.57) and CD_4_^+^ count ≤ 200 cells/μl (AOR: 4.77; 95% CI: 1.23, 18.45) were predictors of leucopenia (Table 4).

**Table 2:** Predictors of anemia

Variables	Anemia		COR (95% CI)	*P*- value	AOR (95% CI)	*P*-value
	Yes *n* (%)	No *n* (%)				
Age						
18–29	35 (58.3)	25 (41.7)	1		1	
30–39	39 (47.0)	44 (53.0)	0.63 (0.32–1.24)	0.181	0.58 (0.27–1.23)	0.157
40–49	11 (47.8)	12 (52.2)	0.65 (0.25–1.72)	0.390	0.73 (0.25–2.12)	0.563
≥ 50	4 (66.7)	2 (33.3)	1.43 (0.24–8.41)	0.693	1.79 (0.23–13.68)	0.571
Sex						
F	55 (52.9)	49 (47.1)	1.12 (0.61–2.07)	0.711		
M	34 (50.0)	34 (50.0)	1			
Resident						
Urban	75 (49.0)	78 (51.0)	1		1	
Rural	14 (73.7)	5 (26.3)	2.91 (1.00–8.48)	0.050	4.67 (1.44–15.14)	0.010
BMI						
< 18.5	23 (52.3)	21 (47.7)	0.73 (0.18–2.95)	0.659		
18.5–24.99	60 (50.8)	58 (69.2)	0.69 (0.18–2.57)	0.580		
> 25	6 (60.0)	4 (40.0)	1			
HAART status						
Yes	70 (49.3)	72 (50.7)	1		1	
No	19 (63.3)	11 (36.7)	1.77 (0.79–4.00)	0.165	1.87 (0.71–4.91)	0.203
Opportunistic infection						
Yes	7 (43.8)	9 (56.2)	0.70 (0.25–1.98)	0.503		
No	82 (52.6)	74 (47.4)	1			
Intestinal parasite						
Yes	8 (61.5)	5 (38.5)	1.54 (0.48–4.91)	0.465		
No	81 (50.9)	78 (49.1)	1			
Malaria infection						
Yes	52 (60.5)	34 (39.5)	2.02 (1.10–3.72)	0.023	2.42 (1.16–5.04)	0.018
No	37 (43.0)	49 (57.0)	1		1	
Parasitic density of malaria						
1–999	12 (57.1)	9 (42.9)	1			
1000–9999	26 (60.5)	17 (39.5)	0.76 (0.22–2.59)	0.664		
≥ 10,000	14 (63.6)	8 (36.4)	0.87 (0.30–2.53)	0.804		
CD_4_^+^ count (cells/μl)						
≤ 200	18 (69.2)	8 (30.8)	1.62 (0.61–4.30)	0.328	1.34 (0.45–3.94)	0.596
200–499	35 (41.7)	49 (58.3)	0.52 (0.26–1.00)	0.051	0.37 (0.17–0.77)	0.009
≥ 500	36 (58.1)	26 (41.9)	1		1	

*COR-Crude odd ratio, AOR-Adjusted odd ratio, CI-Confidence interval, 1-indicator and Statistical significant at *p* < 0.05

**Table 3:** Predictors of thrombocytopenia

Variables	Thrombocytopenia		COR (95% CI)	*P*-value	AOR (95% CI)	*P*-Value
	Yes *n* (%)	No *n* (%)				
Age						
18–29	19 (31.7)	41 (68.3)	1			
30–39	32 (38.6)	51 (61.4)	1.35 (0.67–2.72)	0.397		
40–49	8 (34.5)	15 (65.5)	1.15 (0.42–3.17)	0.786		
≥ 50	2 (33.3)	4 (66.7)	1.08 (0.18–6.41)	0.933		
Sex						
F	37 (35.6)	67 (64.4)	1.05 (0.56–1.99)	0.868		
M	24 (35.3)	44 (64.7)	1			
HAART status						
Yes	45 (31.7)	97 (68.3)	1		1	
No	16 (53.3)	14 (46.7)	0.41 (0.18–0.90)	0.027	1.46 (0.59–3.59)	0.407
Opportunistic infection						
Yes	5 (31.2)	11 (68.8)	0.81 (0.27–2.45)	0.712		
No	56 (35.9)	100 (64.1)	1			
Malaria infection						
Yes	51 (59.3)	35 (40.7)	11.07 (5.04–24.33)	<0.001	9.79 (4.33–22.17)	<0.001
No	10 (11.6)	76 (88.4)	1		1	
Malaria parasitic density						
1–999	12 (57.1)	9 (42.9)	1			
1000–9999	26 (60.5)	17 (39.5)	1.15 (0.39–3.30)	0.799		
≥ 10000	13 (59.1)	9 (40.9)	1.08 (0.32–3.64)	0.897		
CD_4_^+^ count (cells/μl)						
≤ 200	16 (61.5)	10 (38.5)	3.36 (1.29–8.71)	0.013	2.69 (0.90–8.05)	0.076
200–499	25 (29.8)	59 (70.2)	0.89 (0.44–1.80)	0.747	0.79 (0.33–1.80)	0.589
≥ 500	20 (32.3)	42 (67.7)	1		1	

*COR-Crude odd ratio, AOR-Adjusted odd ratio, CI-Confidence interval, 1-indicator and statistically significant at *p* < 0.05

**Table 4:** Predictors of leucopenia

Variables	Leucopenia		COR (95% CI)	*P*-value	AOR (95% CI)	*P*-Value
	Yes *n* (%)	No *n* (%)				
Sex						
F	41 (39.4)	63 (60.6)	0.98 (0.53–1.84)	0.970		
M	27 (39.7)	41 (60.3)	1			
Age						
18–29	23 (38.3)	37 (61.7)	1			
30–39	35 (42.2)	48 (57.8)	1.17 (0.59–2.31)	0.645		
40–49	7 (30.4)	16 (69.6)	0.70 (0.25–1.97)	0.504		
≥ 50	3 (50.0)	3 (50.0)	1.61 (0.23–8.65)	0.580		
HAART status						
Yes	56 (39.4)	86 (60.6)	1			
No	12 (40.0)	18 (60.0)	1.02 (0.46–2.28)	0.954		
Opportunistic infection						
Yes	5 (31.2)	11 (68.8)	0.67 (0.22–2.02)	0.479		
No	63 (40.4)	93 (59.6)	1			
Malaria infection						
Yes	37 (43.0)	49 (57.0)	1.34 (0.73–2.47)	0.350		
No	31 (36.0)	55 (64.0)	1			
Malaria parasitic density (parasite/μl)						
1–999	13 (61.9)	8 (38.1)	1		1	
1000–9999	20 (46.5)	23 (53.3)	0.53 (0.18–1.55)	0.250	0.55 (0.18–1.69)	0.303
≥ 10000	4 (18.2)	18 (81.8)	0.14 (0.34–0.55)	0.005	0.13 (0.03–0.57)	0.007
CD_4_^+^ count (cells/μl)						
≤ 200	16 (61.5)	10 (38.5)	7.12 (2.58–19.61)	<0.001	4.77 (1.23–18.45)	0.023
200–499	25 (29.8)	59 (70.2)	3.11 (1.47–6.57)	0.003	2.85 (0.94–8.62)	0.064
≥ 500	20 (32.3)	42 (67.7)	1		1	

*COR-Crude odd ratio, AOR-Adjusted odd ratio, CI-Confidence interval, 1- indicator and statistically significant at *p* < 0.05

## Discussion

The current study has attempted to provide information on the effect of malaria on the hematological profiles of PLWHA. The major observations are: the prevalence of anemia, thrombocytopenia and leucopenia were higher in malaria infected participant than malaria non-infected participants. The mean hematological profiles were lower in participants with malaria infection than without malaria infection. Residence, malaria infection and CD_4_^+^ count were identified as predictors for anemia. Malaria infection was significantly associated with thrombocytopenia, whereas malaria parasitic density and CD_4_^+^ count were significantly associated with leucopenia.

In this study the prevalence of anemia was higher (60.5%) in malaria and HIV co-infected participants than those HIV mono-infected participants (43.0%). This finding was in agreement with other studies [17, 21–23]. However, the prevalence of anemia in malaria and HIV co-infected participants was lower as compared to studies conducted in Ghana (2012), Nigeria (2006) and Gondar (2013) which reported 97.1%, 66.7% and 71.3%, respectively [17, 21, 22]. The difference might be due to variation in methods according to sample size they use a small sample size and clinical condition like HAART status and immune status of the participants. Anemia due to malaria infection can occur through different mechanisms; include RBC lysis, organ sequestration, phagocytosis of uninfected and infected RBCs, and dyserythropoiesis [3].

The second prevalent hematological abnormality in malaria and HIV co-infected study participants was thrombocytopenia (59.3%) and in HIV mono-infected study participants (11.6%). This shows there is a significant association between malaria infection and thrombocytopenia, which is supported by a study done in Nigeria (60%) (2006) [21]. The possible causes of thrombocytopenia in malaria infection was increased sequestration and highly elevated levels of platelet bound immunoglobulin that leads to increased peripheral destruction [4].

Among malaria and HIV co-infected participants, 43.0% had leucopenia and among HIV mono-infected participants 36.0% had leucopenia. There was no statistically significant difference between the two groups. In contrary, the study done in Nigeria (2006) reported that the occurrence of leucopenia were more than two times higher in malaria and HIV co-infected participants than HIV mono-infected participants [21]. This difference might be due to variation in the method used for investigation of hematological profiles, In Nigeria the white cell counting was done manually where as in the current study cell count was done using Cell Dyne 1800 hematology analyzer. The cut off values they used for the leucopenia was ≤ 3 × 10^9^/l but in our case leukopenia was defined as WBC count < 4.0 × 10^9^/l. Sometimes, reduction in the leukocyte counts is attributed to hypersplenism or sequestration in the spleen rather than actual depletion [5].

Our result showed that the prevalence of *Plasmodium falciparum* (94.2%) was higher than the prevalence of *Plasmodium vivax* (3.5%) in malaria and HIV co-infected participants. It is consistent with the studies conducted in Gondar (2013) and Nigeria (2006) [17, 21] but contradicts to the study done in India (2012) [24]. This difference might be due to geographical and climatic variations of the study area. *Plasmodium falciparum* is highly prevalent in Sub-Saharan country than Asia and Latin America [25].

The mean value of Hgb, HCT, platelet and lymphocyte count were lower in malaria and HIV co-infected participants than malaria non-infected participants. This finding is consistent with the previous studies done in Nigeria (2006 and 2013) [21, 26]. However, this result contradicts to the study from Cameroon in 2012 [27]. This difference might be due to a small number of study participants involved in Cameroon study. In contrary, in our study there was no significant difference in the mean value of RBC, MCV, MCH and MCHC in malaria infected and non-infected PLWHA. This finding is supported by a study done in Cameroon (2012) [27]. This might be due to normocytic normochromic (NCNC) nature of anemia in both malaria and HIV infections.

Multivariable logistic regression analysis of this study showed that: those study participants live in rural area were more likely to be anemic than participants live in urban areas. It might be due to the type of food the rural community consume, lack of frequent follow up and other factors which could cause anemia. Malaria infected PLWHA were two times more likely to be anemic than malaria non-infected PLWHA. This result is consistent with the study done in Nigeria (2012) [23]. Likewise, malaria infected study participants were ten times more likely to be thrombocytopenic than malaria non-infected PLWHA.

Participants who had CD_4_^+^ count ≤ 200 cells/μl were nearly five times more likely to have leucopenia than those who had CD_4_^+^ count ≥ 500 cells/μl PLWHA. This finding is consistent with other studies done in India (2012), Brazil (2011) and Uganda (2014) [28–30]. Another predictor of leucopenia in this study was malaria parasitic density. Those study participants who had a higher malaria parasitic density were 13% less likely to be leucopenic than those having the lowest parasitic density. However, in contrast to this a study from Cameroon (2012) reported that parasite density is not significantly associated with any hematological parameters [27].

Malaria and HIV are highly prevalent infection in the developing countries, especially in sub-Saharan Africa. They cause hematological abnormalities which decrease the immune response and increase adult mortality. Therefore, assessment of hematological abnormalities in malaria HIV co-infection individuals has remarkable benefit to prevent HIV malaria co-morbidity [31].

In general, this study was a comparative cross sectional study which tried to identify independent factors of hematological profiles in HIV and malaria co infected individuals. The limitations of the study are: first this study is limited by its cross sectional design, not longitudinal, preventing assessment of cause and effect relationship. Second, we did not consider the malaria seasonality and species identification using PCR.

## Conclusions

Findings suggest that the prevalence of anemia and thrombocytopenia were significantly higher in the malaria and HIV coinfected patients than the HIV mono-infected patients. Mean values of hematological profiles were significantly different in the two groups. Future prospective studies with larger sample size from other settings are needed to substantiate the findings.
